# Supplementary material for: Novel Protein-Based Vaccine against Self-Antigen Reduces the Formation of Sporadic Colon Adenomas in Mice
Source: Cancers (Basel). 2021 Feb 17;13(4):845. doi: 10.3390/cancers13040845 (PMC7923075; doi:10.3390/cancers13040845)
Supplement: Supplementary file 1 [file cancers-13-00845-s001.pdf]

cancers

Supplementary Materials: Novel Protein-Based Vaccine Against Self-Antigen Reduces the Formation of Sporadic Colon Adenomas in Mice

Elodie Belnoue, Alyssa A. Leystra, Susanna Carboni, Harry S. Cooper, Rodrigo T. Macedo, Kristen N. Harvey, Kimberly B. Colby, Kerry S. Campbell, Lisa A. Vanderveer, Margie L. Clapper and Madiha Derouazi

A

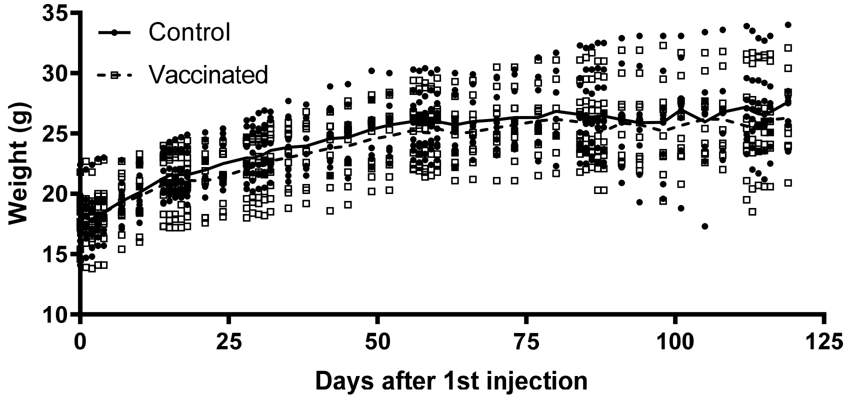

B

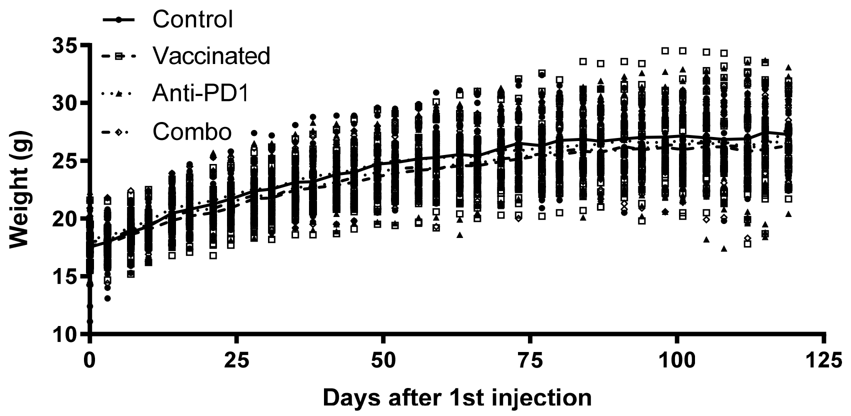

Figure S1. Body weights of mice during an assessment of the KISIMA-Mash2 vaccine with or without anti-PD-1 treatment. (**A**) Weekly body weights of APC$^{+/Min-FCCC}$ mice during an assessment of the KISIMA-MASH2 vaccine or (**B**) KISIMA-MASH2 vaccine and anti-PD-1 combination therapy.

Table S1. Characteristics of mice enrolled on immunogenicity study at time of first treatment.

Table	Baseline Tumor Status	Mice (n)	Age (Days)*	Weight (g)*
Buffer	Tumor-Free	8	43 ± 4 (37–48)	18.1 ± 2.5 (14.1–22.4)
	Tumor-Bearing	0	NA	NA
	Not Determined	2	36 ± 2 (31–46)	17.3 ± 1.7 (16.1–18.5)
	All	8	41 ± 5 (34–48)	18.0 ± 2.4 (14.1–22.4)
Vaccine	Tumor-Free	6	43 ± 4 (39–46)	18.4 ± 2.2 (15.4–21.8)
	Tumor-Bearing	1	42	19.5
	Not Determined	2	37 ± 1 (36–37)	16.2 ± 2.2 (14.6–17.7)
	All	9	41 ± 4 (36–46)	18 ± 2.2 (14.6–21.8)

N/A—Not applicable; *Mean ± standard deviation (range)

Table S2. Characteristics of mice enrolled on efficacy study at time of first treatment.

Treatment Arm	Baseline Tumor Status	Mice (n)	Age (Days)*	Weight (g)*
Buffer	Tumor-Free	9	42 ± 4 (38–49)	18.8 ± 1.4 (16.1–20.7)
	Tumor-Bearing	1	39	18.7
	Not Determined	16	37 ± 5 (31–46)	16.6 ± 2.4 (11.1–19.9)
	All	26	39 ± 5 (31–49)	17.5 ± 2.3 (11.1–20.7)
Vaccine	Tumor-Free	10	41 ± 4 (38–49)	18.5 ± 1.0 (16.9–19.5)
	Tumor-Bearing	1	45	21.6
	Not Determined	11	36 ± 3 (33–41)	16.5 ± 1.1 (15.5–19.4)
	All	22	39 ± 4 (33–49)	17.6 ± 1.7 (15.5–21.6)
anti-PD-1	Tumor-Free	14	44 ± 4 (38–49)	19.0 ± 1.3 (17.1–22.3)
	Tumor-Bearing	0	N/A	N/A
	Not Determined	10	37 ± 4 (33–45)	17.2 ± 1.6 (14.7–19.5)
	All	24	41 ± 5 (33–49)	18.3 ± 1.7 (14.7–22.3)
Combination	Tumor-Free	9	42 ± 5 (35–49)	19.2 ± 1.5 (16.9–21.5)
	Tumor-Bearing	1	49	18.2
	Not Determined	10	38 ± 4 (31–45)	16.8 ± 1.5 (14.2–18.3)
	All	20	41 ± 5 (31–49)	17.9 ± 1.9 (14.2–21.5)

N/A—Not applicable; *Mean ± standard deviation (range).

Table S3. MHC class I and class II predicted epitopes in the peptide pools.

Predicted By:	Epitope Sequence	MHC Molecule	Algorithm Score				Peptide Pool n
		Allele	Syfpeithi	NetMHC	Rankpep	NetMHCpan	
4 algorithms	VNLGFQAL	Kb	21	0.02	9.719	0.017	1
	AQGLNASLM	Db	27	0.08	18.056	0.054	2
	SAVEYIRA	Kb	14	1.6	7.013	1.164	3
	LVNLGFQAL	Kb	11	1.8	11.246	1.365	1
3 algorithms	SAVEYIRAL	Kb	19	0.17	-	0.054	3
	ASPASASL	Kb	13	0.8	-	0.47	3
	RNRVKLVNL	Kb	13	0.7	-	0.524	1
	ASLMDGGAL	Db	16	0.5	-	0.609	2
	SASPASASL	Db	16	-	12.867	0.689	3
	GAQGLNASLM	Db	15	0.6	-	0.691	2
	KKLSKVETL	Db	18	-	11.331	1.031	1
	VKLVNLGFQAL	Db	-	1.1	29.589	1.722	1
2 algorithms	LSPMEQELL	Db	18	-	-	0.364	3
	AGAQGLNASLM	Db	-	1.5	-	0.392	2
	VHFPPHPV	Db	-	-	3.111	0.583	2
	AVEYIRAL	Kb	12	-	-	0.797	3
	YIRALQRL	Kb	11	-	-	0.902	3
	TSPSPDRL	Kb	12	-	-	1.435	3
	STSPSPDRL	Db	15	-	-	1.585	3
	LSPMEQEL	Kb	13	-	-	1.825	3
	EYIRALQRL	Db	16	-	-	1.849	3
	AAPELVAGA	Db	10	-	-	1.97	2
	RSAVEYIRAL	Kb	13	1.6	-	-	3
	ASTSPSPDRL	Db	17	-	9.734	-	3
	GELSPMEQEL	Db	16	-	10.191	-	3
	DGGALPRLM	Db	14	-	10.236	-	2
	HGGANKKLSK	Db	14	-	14.062	-	1
	KLVNLGFQAL	Db	14	-	9.65	-	1
	EHFSCAAPEL	Db	13	-	4.96	-	2
	FSCAAPELV	Db	13	-	4.307	-	2
	GANKKLSKV	Db	13	-	5.138	-	1
	RERNRVKL	Kb	12	-	9.191	-	1
	RQASPELL	Kb	12	-	9.754	-	2

	FSCAAPEL	Kb	11	-	13.66	-	2
Class II (1 algorithm)	CAQPSASPA	I-Ab	-	-	17.44	-	3
	VEYIRALQR	I-Eb	-	-	16.102	-	3
	HDAVRAALA	I-Ab	-	-	12.378	-	3
	VAGACAARR	I-Ab	-	-	12.093	-	2
	LLTPATPPS	I-Ab	-	-	10.022	-	3

Table S4. Intraepithelial and stromal CD3+ lymphocytes as a ratio of cells per dysplastic area and cells per lesion.

Cell Type	Tissue Compartment	Buffer*	Vaccine + Anti-PD1*	*p*-value†
CD3+ cells/mm²‡	Intraepithelial	96 ± 22	179 ± 23	0.02
CD3+ cells/mm²‡	Stromal	746 ± 110	733 ± 139	0.413
CD3+ cells/adenoma	Intraepithelial	38 ± 14	104 ± 3	0.065
CD3+ cells/adenoma	Stromal	249 ± 60	270 ± 63	0.39

*Mean ± Standard Error of the Mean; †Two-sided Student T-test; ‡Number of cells normalized to the area (mm^2) of dysplasia in each animal.

Table S5. Intraepithelial and stromal CD3+ cells per microadenoma.

Tissue Compartment	Buffer*	Vaccine + Anti-PD1*	*p*-value†
Intraepithelial	0.5 ± 0.3	1 ± 0.7	0.537
Stromal	19 ± 8	11 ± 6	0.471

*Mean ± Standard Error of the Mean; †Two-sided Student T-test.

Text S1

Animals

$Apc^{+/Min-FCCC}$ mice were maintained on autoclaved Rodent Breeder Diet (LabDiet #5013) ad libitum until colonoscopy, and then switched to autoclaved 2018 Teklad 18% Protein/Extruded Global Rodent Diet (Envigo #2018SX) for the duration of the study. Animals were housed in individually ventilated cages with free access to water in rooms maintained at 70 ± 2 °F and 40–70% relative humidity with a 12-hour light/dark cycle (lights on at 6:00 h and lights off at 18:00 h).

Timing of Therapy and Sample Collection

All treatments were administered between 12:30 –15:00 h on the scheduled day. The first three blood draws for each mouse were completed between 13:00–15:00 h, and prior to performing any injections on the same day. Blood draws performed at the end of the study prior to necropsy were completed between 7:30–9:30 h. Necropsies were performed between 8:00–15:00 h.

Copyright: © 2021 by the authors. Licensee MDPI, Basel, Switzerland. This article is an open access article distributed under the terms and conditions of the Creative Commons Attribution (CC BY) license (http://creativecommons.org/licenses/by/4.0/).
